# Duane retraction syndrome in a Nigerian child

**DOI:** 10.11604/pamj.2014.19.96.4641

**Published:** 2014-09-26

**Authors:** Olusola Oluyinka Olawoye, Bolutife Ayokunnu Olusanya, Aderonke Mojisola Baiyeroju

**Affiliations:** 1 Department of Ophthalmology, University College Hospital, University of Ibadan, Ibadan, Nigeria

**Keywords:** Duane syndrome, West Africa, child, squint, eye movement

## Abstract

We report a case of a four year old Nigerian girl who presented to the paediatric unit of our eye clinic with complaints of a squint on looking to the right side and reduction in the size of the right eye when looking to the left. On examination, she had right exotropia in the primary position of gaze. There was limitation of abduction and widening of the palpebral fissure of the right eye on right gaze. On left gaze there was narrowing of the palpebral fissure of the right eye with marked limitation of adduction and an upshoot, or occasionally a downshoot, of the eyeball. This report demonstrates that Duane's syndrome occurs in West Africa. Therefore, detailed examination of all patients with squints is important to ensure accurate diagnosis and appropriate management of the patient's condition.

## Introduction

Duane retraction syndrome (DRS) is a congenital ocular motility disorder characterized by moderate to severe limitation of abduction and varying degrees of adduction deficit, with associated palpebral fissure narrowing and globe retraction on adduction. [1] It is the commonest form of the Congenital Cranial Dysinnervation Disorders. [2] DRS is due to mal-development of certain centers in the brainstem responsible for the control of the extraocular muscles. There is often hypoplasia or absence of the sixth cranial nerve which normally supplies the lateral rectus with abnormal innervation of the lateral rectus by a branch from the ipsilateral third cranial nerve [2].

DRS accounts for 1-5% of all cases of strabismus. [3] It affects one eye in 85% of cases [3], most often the left eye (72%) and it is more prevalent in females (60%).[4] Bilateral cases are usually associated with asymmetrical involvement. This syndrome oftentimes is sporadic; however, numerous cases of familial transmission mostly bilateral with an autosomal dominant pattern have been reported. In 70% of patients, DRS is an isolated disorder. But there are systemic associations which may include Goldenhar's syndrome, Klippel–Feil syndrome, Okihiro (Duane radial ray) syndrome, Holt-Oram syndrome and Wildervanck syndrome. [4] In addition, maternal thalidomide ingestion, fetal alcohol syndrome, and oculocutaneous albinism have been associated with DRS. [5] A diagnosis of Duane retraction syndrome may easily be missed because of its relative rarity and because of the complex nature of its clinical features. We report a case of Duane syndrome from West Africa. To the best of our knowledge this is the first report from this region.

## Patient and observation

A four year old girl presented to our clinic with a history of a squint since birth that occurs when she looks to the right side. Her mother also noticed that the right eye appears smaller when she looks to the left. She had no history of double vision when looking straight ahead. There was no history of antecedent trauma, headache, or vomiting. Her pregnancy and delivery were uneventful; and her development was normal. There was no major illness in early childhood. She had no other systemic complaints. All other family members were well.

**Figure 1:**
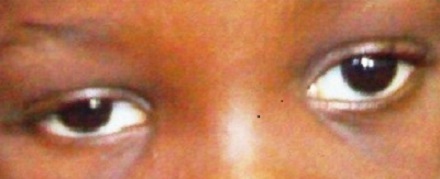
Right exotropia in primary gaze position

**Figure 2:**
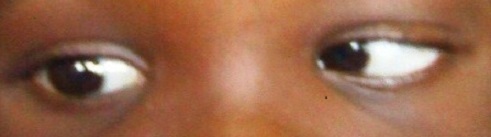
Limitation of abduction of the right eye with widening of the right palpebral fissure and full adduction of the left eye

**Figure 3:**
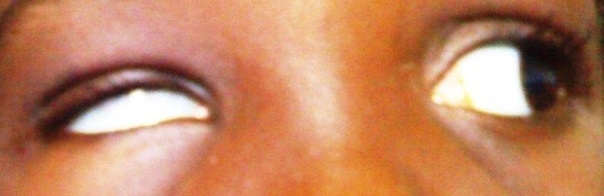
Narrowing of the right palpebral fissure with limitation of adduction and an upshoot of the right eye

**Figure 4:**
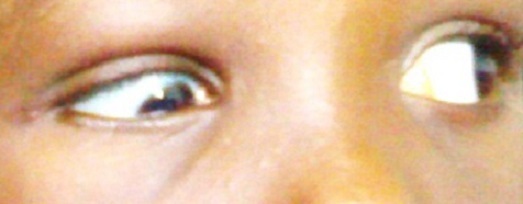
A down shoot of the right eye with limitation of adduction and narrowing of the palpebral fissure

On examination, she had a visual acuity of 6/6 in each eye with normal anterior and posterior segments bilaterally. There was 12 prism diopters of right exotropia in the primary position (Figure 1) and she did not have an abnormal head posture. Her palpebral fissures were normal and equal on both sides in the primary position of gaze. On right gaze, however, there was slight widening of the right palpebral fissure, moderate limitation of abduction of the right eye and normal adduction of the left eye (Figure 2). On left gaze, there was narrowing of the right palpebral fissure with marked limitation of adduction of the right eye. An upshoot or occasionally, a down shoot of the eye ball was observed on attempted maximal adduction of the right eye (Figure 3, Figure 4). There was normal abduction of the left eye.

Forced duction test (performed under sedation with Choral hydrate) revealed no restriction of lateral and medial movement of the right eye. There was no clinical evidence of amblyopia and she had no refractive error. No abnormality was detected on systemic examination. Our findings were consistent with a weakness of the right lateral rectus muscle and a restriction of adduction with globe retraction, presumably, as a result of co-contraction of both the medial and lateral rectus muscles on left gaze. We, therefore, made a diagnosis of Right Duane Retraction Syndrome. She was managed conservatively since there was no diplopia in primary and reading position, and she did not have an abnormal head posture. Her mother was educated about the condition and she was told that she should learn to turn her head and not her eyes when looking to the sides.

## Discussion

Duane retraction syndrome (DRS) has been a well-recognized clinical entity for more than a century. Jakob Stilling (1887) [6], Siegmund Türk (1896) [7] and Alexander Duane (1905) [8] were the first to describe this particular form of strabismus. In European literature, the retraction syndrome is appropriately referred to as the Stilling–Turk–Duane syndrome. It is rare in the general population with an incidence of about 0.1%. [4] Prior reviews of DRS comprise mostly of unilateral cases occurring predominantly in females, as in our patient. This observation led to the hypothesis that the gene was partly sex-linked. [4] Our patient had a unilateral right sided involvement. Left side predominance has been cited in all the studies on DRS over the last century. [4] When all major studies were pooled together, in a total number of 835 patients, 59% were left sided, 23% were right sided and 18% were bilateral [4].

Although our patient did not have any clinical feature suggestive of amblyopia, amblyopia has been reported to range from 3% to 25%, with a weighted average of 14%. [4] Amblyopia is mainly due to strabismus and not to anisometropia. [3] Absence of diplopia as in this case, may be because of regional suppression when she looks in the field of action of the right lateral rectus [9]. A possible alternative diagnosis in our patient is a Congenital Fibrosis of the extraocular muscles affecting the lateral rectus. This might explain the restriction of adduction, the narrowing of the palpebral fissure as well as the upshoots and down shoots but the negative forced duction test does not support this diagnosis in our patient. Another differential diagnosis is intermittent exotropia in view of the history that the squint was not present all the time; however, palpebral fissure narrowing and upshoots or down shoots are not features of intermittent exotropia. Therefore, we considered Duane Retraction syndrome to be the most likely diagnosis in this patient.

In 1974, Huber [10] classified DRS into the three types: Duane 1, Duane 2, and Duane 3. Type 1 is characterized by marked limitation or complete absence of abduction with associated esotropia, and is the most common type. Type 2 is characterized by marked limitation of adduction and exotropia of the involved eye, and is the least common; while type 3 is characterized by marked limitation of both adduction and abduction and is often associated with a straight eye in primary position of gaze. We believe the clinical features in our patient are suggestive of DRS type 2. The aetiopathogenesis of DRS has been extensively studied. Huber [10] suggested, based on electromyography (EMG) findings, that a paradoxical anomalous innervation of the lateral rectus muscle from the oculomotor (third cranial nerve) nucleus is the underlying cause in all three types of DRS. This causes the lateral rectus muscle to co-contract with the medial rectus on attempted adduction with resultant retraction of the globe and narrowing of the palpebral aperture.

## Conclusion

Cases of DRS have been reported from almost all areas of the world, and no particular race or ethnic group has been identified to have a predilection for the syndrome. This, however, is the first report of this syndrome from the West African region to the best of our knowledge. It is important to document that this syndrome also occurs in this region since accurate diagnosis is very important in excellent patient management.
